# The transcription factor VAX1 in VIP neurons of the suprachiasmatic nucleus impacts circadian rhythm generation, depressive-like behavior, and the reproductive axis in a sex-specific manner in mice

**DOI:** 10.3389/fendo.2023.1269672

**Published:** 2023-12-22

**Authors:** Brooke M. Van Loh, Alexandra M. Yaw, Joseph A. Breuer, Brooke Jackson, Duong Nguyen, Krystal Jang, Fabiola Ramos, Emily V. Ho, Laura J. Cui, Dominique L. M. Gillette, Lorenzo F. Sempere, Michael R. Gorman, Karen J. Tonsfeldt, Pamela L. Mellon, Hanne M. Hoffmann

**Affiliations:** ^1^ Department of Animal Science and the Reproductive and Developmental Sciences Program, Michigan State University, East Lansing, MI, United States; ^2^ Department of Obstetrics, Gynecology, and Reproductive Sciences and Center for Reproductive Science and Medicine, University of California, San Diego, La Jolla, CA, United States; ^3^ Department of Radiology and Precision Health Program, Michigan State University, East Lansing, MI, United States; ^4^ Department of Psychology, University of California, San Diego, La Jolla, CA, United States; ^5^ Center for Circadian Biology, University of California, San Diego, La Jolla, CA, United States

**Keywords:** ventral anterior homeobox 1, vasoactive intestinal peptide, arginine vasopressin, suprachiasmatic nucleus, reproduction, premenstrual disorders, circadian rhythm, cortisol

## Abstract

**Background:**

The suprachiasmatic nucleus (SCN) within the hypothalamus is a key brain structure required to relay light information to the body and synchronize cell and tissue level rhythms and hormone release. Specific subpopulations of SCN neurons, defined by their peptide expression, regulate defined SCN output. Here we focus on the vasoactive intestinal peptide (VIP) expressing neurons of the SCN. SCN VIP neurons are known to regulate circadian rhythms and reproductive function.

**Methods:**

To specifically study SCN VIP neurons, we generated a novel knock out mouse line by conditionally deleting the SCN enriched transcription factor, Ventral Anterior Homeobox 1 (Vax1), in VIP neurons (Vax1^Vip^; Vax1^fl/fl^:Vip^Cre^).

**Results:**

We found that Vax1^Vip^ females presented with lengthened estrous cycles, reduced circulating estrogen, and increased depressive-like behavior. Further, Vax1^Vip^ males and females presented with a shortened circadian period in locomotor activity and *ex vivo* SCN circadian period. On a molecular level, the shortening of the SCN period was driven, at least partially, by a direct regulatory role of VAX1 on the circadian clock genes *Bmal1* and *Per2*. Interestingly, Vax1^Vip^ females presented with increased expression of arginine vasopressin (*Avp*) in the paraventricular nucleus, which resulted in increased circulating corticosterone. SCN VIP and AVP neurons regulate the reproductive gonadotropin-releasing hormone (GnRH) and kisspeptin neurons. To determine how the reproductive neuroendocrine network was impacted in Vax1^Vip^ mice, we assessed GnRH sensitivity to a kisspeptin challenge *in vivo*. We found that GnRH neurons in Vax1^Vip^ females, but not males, had an increased sensitivity to kisspeptin, leading to increased luteinizing hormone release. Interestingly, Vax1^Vip^ males showed a small, but significant increase in total sperm and a modest delay in pubertal onset. Both male and female Vax1^Vip^ mice were fertile and generated litters comparable in size and frequency to controls.

**Conclusion:**

Together, these data identify VAX1 in SCN VIP neurons as a neurological overlap between circadian timekeeping, female reproduction, and depressive-like symptoms in mice, and provide novel insight into the role of SCN VIP neurons.

## Introduction

The circadian system is responsible for coordinating circadian and time-of-day signals throughout the body. On a cellular level, circadian rhythms are produced by the molecular clock, a transcription/translation feedback loop that generates 24-hour cell autonomous rhythms (1–3). Alterations in molecular clock function can lead to changes in circadian behaviors and fertility, as shown in transgenic mouse models with a loss of *Bmal1*, a gene required for molecular circadian rhythm generation (4–8). Along with proper molecular clock function, the suprachiasmatic nucleus (SCN), located in the ventral hypothalamus, is the central pacemaker of the brain and serves to coordinate external timing signals and physiological processes throughout the body. Importantly, the SCN requires a finely balanced neuropeptide expression to maintain circadian rhythms (9). Correct levels of vasoactive intestinal peptide (VIP), along with other neuropeptides such as arginine vasopressin (AVP), somatostatin, and gastric releasing peptide, are required for SCN output controlling synchrony both within and downstream of the SCN, including behavioral and tissue level circadian rhythms. Both the molecular clock and neuropeptide expression must function correctly to maintain strong and synchronized circadian rhythms (10–13). SCN VIP-expressing neurons play an important role in aligning SCN function to the time of day by relaying photic information from the optic nerve to generate synchrony among SCN neurons and SCN output (14, 15).

One of the well-established downstream effects of SCN VIP neurons is the regulation of neuroendocrine function, including the regulation of gonadal sex steroids needed for female fertility (6, 16–19). In males, the role of VIP neurons with regard to reproductive function is less clear. It has been suggested that VIP may be involved in testicular health (20, 21), but no reproductive phenotype has been reported in any *Vip* knock-out mouse line to our knowledge. Additional studies have demonstrated that impaired SCN function or disrupted circadian rhythms in the SCN or periphery do not, or only modestly, disrupt male fertility, supporting the idea that male fertility is more robust than female fertility and often resilient to circadian disruption (22–26). In contrast, female fertility relies on precise synchronization between hormone release and peripheral tissue function (24, 27, 28). To regulate the reproductive axis, VIP neurons project directly and indirectly through SCN AVP neurons and kisspeptin neurons (29–32) to gonadotropin-releasing hormone (GnRH) neurons. GnRH is released through both pulse and surge modes into the median eminence, promoting the pituitary to release luteinizing hormone (LH) and follicle-stimulating hormone (FSH). The timing of the LH surge is particularly important in females at both the level of the hypothalamus and the ovary for optimal ovulation (33, 34). This surge requires coordinated input to GnRH neurons from both the SCN and kisspeptin neurons. In the ovary, the molecular clock is required in ovarian theca cells to increase LH receptor expression around the time of day of the LH surge, facilitating ovulation (35).

Given their importance for both circadian timekeeping and reproductive health, the role of VIP neurons in the SCN is an important area of investigation in the context of female reproduction. Previous work has shown that changes in VIP alter circadian rhythms in mice (36–38), with full-body VIP knock-out female mice having disruptions in both the circadian and reproductive systems, demonstrating the importance of VIP in both processes (19). Such changes in VIP can also negatively impact reproductive hormone release through dysregulation of the required neurocircuitry for LH and FSH release (39–41), which can, in turn, lead to dysregulation of female reproductive sex steroids (42), an imbalance that can have negative effects on both reproductive function and mood (43, 44). Evidence supports that disrupted circadian rhythms and changes in estrogen and progesterone might be contributing factors to depressive symptoms in humans (41, 45). Together this evidence indicates a potential shared origin between circadian deregulation, reproductive deficits, and mood changes at the level of VIP neurons.

In this study, our goal was to determine if abnormal SCN VIP neuron function causes disruption in circadian timekeeping, fertility, and mood. We deleted the SCN-enriched transcription factor, Ventral anterior homeobox 1 (VAX1) within the VIP neurons of mice. Our previous work has shown that VAX1 is required for SCN development (46, 47) and maintains a function in late development of the SCN and VIP neurons, where it is required for VIP expression, SCN output and female fertility (25). Due to the specific overlap between VAX1 and VIP in the SCN, shown here, the use of Vax1^flox/flox^:Vip^Cre^ mice provides a model to specifically investigate how weakened, but not ablated, SCN VIP neuron function regulates reproductive function, SCN circadian output, and mood. Identifying the shared genetic underpinnings of the association of reproductive and mood disorders is a required first step towards future development of efficient strategies to improve hormone related mood disorders. We hypothesize that VAX1 in postnatal VIP neurons is required for VIP neuron function, where loss of VAX1 in VIP neurons causes weakened SCN output, leading to female, but not male, subfertility and increased depressive-like symptoms.

## Materials and methods

### Mouse breeding

All animal procedures were performed according to protocols approved by the University of California, San Diego Institutional Animal Care and Use Committee and the Institutional Animal Care and Use Committee of Michigan State University and conducted in accordance with the Guide for the Care and Use of Laboratory Animals (48). Mice were maintained on a light/dark cycle of LD [12 h light, 12 h dark, average light intensity ~150-350 lux within the cage]. Based on the newly proposed light reporting method by (49, 50), we determined the relative perception of light by mice using the mouse α-opics equivalent daylight illuminance (EDI, **Supplementary Figure 1**). We calculated that the α-opics for our experimental mice on LD to be melanopsin = 43.2 ± 16.9 lux, rod = 51.6 ± 19.8 lux, s-cone = 0.03 ± 0.01 lux, and m-cone = 57.1 ± 21.7 lux. Lights ON = ZT0 (6:00), lights OFF = ZT12 (18:00), Zeitgeber time (ZT). Vax1^flox/flox^ (Vax1^tm1c(KOMP)Mbp^, MGI: 5796178) (51), were crossed with mice heterozygous for the Vip^Cre/wt^ allele (JAX #010908). Period2::Luciferase (PER2::LUC) mice were purchased from JAX (strain B6.129S6-Per2tm1Jt/J, JAX #006852) and crossed with the offspring of Vax1^flox/flox^ and Vip^Cre/wt^ mice. We did not detect any significant differences in mice heterozygous for the Vip^Cre/wt^ allele as compared to Vax1^flox/flox^ mice, thus we pooled the Vax1^flox/flox^ and Vip^Cre/wt^ control groups into one control group, referred to as Ctrl. Genotyping primer sequences were as follows: Vax1-wtF: CCAGTAAGAGCCCCTTTGGG, Vax1-floxF: GCCGGAACCGAAGTTCCTA; Vax1-R: CGGATAGACCCCTTGGCATC; CreF: GCATTACCGGTCGTAGCAACGAGTG, CreR: GAACGCTAGAGCCTGTTTTGCACGTTC. Mice were kept on a C57BL/6J background and were screened for germline recombination. Mice with germline recombination were excluded from the studies. VIP-tdTomato mice were generated by crossing Vip^Cre/wt^ mice with Rosa-tdTomato reporter mice (JAX# 007914). Mice were euthanized by cervical dislocation followed by decapitation.

### Wheel-running behavior

Female and male mice aged 8-12 weeks at the start of the experiment were single-housed in cages containing metal running wheels and wheel revolutions were monitored using magnetic sensors. All cages were contained in a light-tight cabinet with programmable lighting conditions and rooms were monitored for temperature and humidity. Sylvania T8 32-Watt 4100K fluorescent bulbs (F032/841/ECO) were used to provide light to the cabinets. Food and water were available *ad libitum* during the entire experiment. After 1-week acclimation to the polypropylene cages (17.8 × 25.4 × 15.2 cm or 33.2 × 15 × 13.2 cm) containing a metal running wheel (11.4 cm diameter or 11 cm diameter, respectively), locomotor activity rhythms were monitored with a VitalView data collection system (Version 4.2, Minimitter, Bend OR) that integrated into 6 minute bins the number of magnetic switch closures triggered by half wheel rotations or full wheel rotations, respectively. Running wheel activity was initially monitored for 2 weeks in a standard 12 h light/12 h dark cycle (LD). Subsequently, mice were monitored for 4 weeks in constant darkness (DD), with wheel running data analyzed from weeks 2-4 (14 days) in DD. Cage changes were scheduled at 3-week intervals. The light intensity varied between 268-369 lux inside the mouse cages. Wheel running activity was analyzed using ClockLab Analysis (ActiMetrics) by an experimenter blind to the experimental group. The circadian period was analyzed by constructing a least-squares regression line through a minimum of 13 daily activity onsets. Daily onset and offset of activity, defined as a period of 5 h of activity following 5 h of inactivity (onset) or a period of 5 h of inactivity following 5 h of activity (offset), were used to calculate the length of the active phase (alpha). Chi^2^ periodograms were generated for periods from 0 to 36 h, with significance set at 0.001. Activity profiles were generated for weeks 2-4 in DD using the estimated chi^2^ periodogram tau for the same time period. Total daily counts for mice on wheels with 2 sensors were calculated over 24 h, during both LD and DD.

### Porsolt forced swim test

Female mice aged 9-13 weeks were single-housed for two weeks on LD, then placed in a transparent 7 by 24 inch cylinder filled 2/3 full with 20-21°C water. Swim tests were completed during the mice active phase and started at ZT13. Mice were recorded in dim red light (5 lux, α-opics: melanopsin = 0.03, rod = 0.20, s-cone = 0, m-cone = 0.54, **Supplementary Figures 1C, F**) for 6 minutes before they were removed from the water, dried with a cloth, and returned to their cages for one hour before euthanasia and blood collection from the abdominal aorta. Videos were scored by an observer blinded to the experimental group using the sampling method where the mouse is determined to be either floating or swimming every 30 seconds for 5 minutes, with the first minute of each video going unscored and serving as an adjustment period. The percent of intervals where the mouse was observed to be floating is reported (52).

### Estrous cyclicity, sperm count, and fertility assessment

For the fertility assessment, virgin 8- to 12-week-old male and female Ctrl, and Vax1^Vip^ mice were housed with opposite-sex Ctrl mice (53, 54). The number of litters and the number of pups per litter were recorded over a period of 4 months, as described previously (54). Estrous cyclicity was monitored by vaginal lavage with 20 µl H_2_O daily between ZT3 and ZT5 for 16-18 days. The lavage solution was dried on a slide and stained with 0.5% methylene blue. Cytology was visually examined and scored. Ovary and uterus weights were collected after euthanasia in diestrus. Following euthanasia in males, testes and epididymis were collected and weighed. Sperm was collected from the epididymis of male mice in M2 media (Sigma #M7167). The epididymis was cut in half and sperm were expelled by gently pressing down on the epididymis and then left in M2 media at room temperature for 15 min. The numbers of total and motile sperm were counted from a 1:10 dilution of the M2 media containing sperm by using a hemocytometer. The second epididymis was cut into small pieces and left 15 minutes at room temperature in M2 media. The solution was homogenized frequently to help liberate the sperm. The solution was filtered using a cell streamer (70 µm, Falcon #352350) and sperm were diluted 1:10 with MQ before counting total number of sperm heads.

### Pubertal onset

Pubertal onset was established by visual inspection of preputial separation (PPS) in males and vaginal opening (VO) in females, as described previously (54). Body weight was recorded daily until pubertal onset was observed.

### Immunohistochemistry staining

Tissues were collected between ZT3 and ZT5 from adult male and proestrus female mice on LD light cycle and fixed overnight at 4°C in 60% ethanol, 10% formaldehyde, and 10% glacial acetic acid. Tissues were washed in 70% ethanol and embedded in paraffin. Single immunohistochemistry on 10 µm coronal brain sections embedded in paraffin was performed as previously described (24). The primary antibody was rabbit anti-VIP (Immunostar #20077, 1:1000, RRID : AB_572270). Sections were incubated in 1:300 secondary anti-rabbit IgG (Vector Laboratories, #BA-1000). Secondary antibodies were purchased from Vector labs, and colorimetric VIP (purple staining) and DAB (brown staining) assays (Vector laboratories) revealed the primary antibodies.

Vip^Cre/wt^:Rosa-tdTomato^+/-^ mice (n=4; 2 female and 2 male) were sacrificed between ZT4-6 at 6 weeks of age and brains immersed in 4% PFA overnight at 4°C. Brains were transferred to 30% sucrose until sectioned 40 μm thick with a cryostat. Sections were stored in cryoprotectant at -80°C. Prior to staining, sections were washed overnight in PBS at 4°C. Sections underwent antigen retrieval for 20 minutes in citrate buffer, followed by a wash and blocking for 30 minutes at room temperature using an Avidin/Biotin blocking kit (Vector Labs) with 5% normal goat serum. Sections were briefly washed and then stained following the protocol for the Mouse on Mouse Basic Immunodetection kit (Vector Labs) using a mouse anti-VAX1 (1:100; Origene RRID : AB_2941013). Slices underwent Vectastain ABC kit (Vector Labs) followed by TSA treatment (Akoya Biosciences) for 10 minutes and finally streptavidin-conjugated secondary (1:200) for 30 minutes. Slices were mounted, air-dried, and coverslipped with Prolong Gold with DAPI (ThermoFisher). Slices were imaged on a Nikon Eclipse Ti2-E using a Lumencor SpectraX LED and acquired using a DS-Qi2 CMOS camera. One SCN section per animal was analyzed using QuPath (55). All image manipulations were applied homogenously to the entire image.

### Multiplex *in situ* hybridization assay

**Figure 1:**
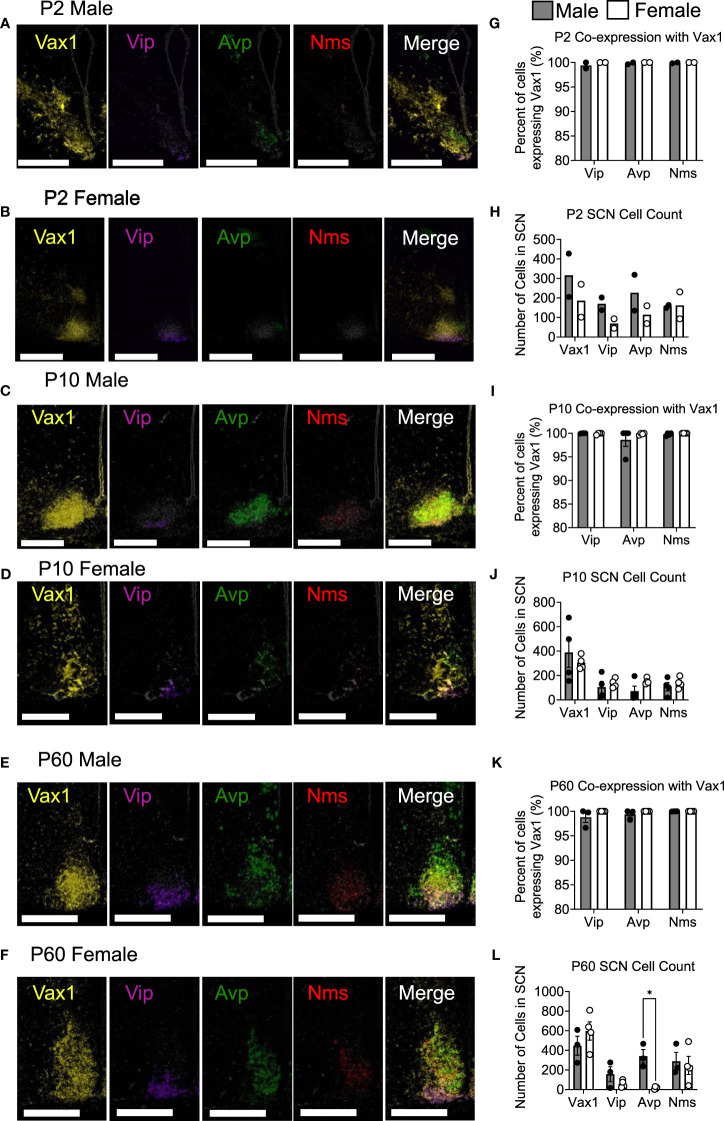
*Vax1* is expressed in SCN *Vip* neurons from the early postnatal period through adulthood in both males and females. RNAscope® ISH representative images **(A–F)**. Images at P2 are displayed with increased contrast, applied to all channels, to compare with P10 and P60. Percent co-expression between *Vax1* and *Vip*, *Avp*, and *Nms*-expressing neurons **(G, I, K)** and cell counts **(H, J, L)** in P2, P10 and P60 males and females at ZT5. Scale bar is 300 µm. Student’s t-test *, p <0.05.

To examine *Vip*, *Avp*, *Nms*, and *Vax1* mRNA when adult male and female hormones are most comparable, brains were collected at ZT4-8 in young mice, adult males, and diestrus females. To examine *Avp*, *Vip*, and *Bmal1* mRNA around the time of the LH surge in Ctrl and Vax1^Vip^ mice, brains were collected at ZT13-16 in proestrus females. Multiplex *in situ* hybridization detection of mouse (*Mus musculus*) mRNAs was performed with RNAscope® LS Multiplex Fluorescent Reagent Kit (Advanced Cell Diagnostics, cat no. 322800) for 3-plex assay in addition to RNAscope® LS 4-Plex Ancillary Kit (Advanced Cell Diagnostics, cat no. 322830) for 4-plex assay following vendor’s standard protocol for FFPE tissue sections with minor modifications. RNAscope® assays were performed on a Leica Bond autostainer as described (56) with the following probes: RNAscope® 2.5 LS Probe – Mm-Arntl (also known as Bmal1) [aryl hydrocarbon receptor nuclear translocator-like (Arntl) transcript variant 1 mRNA, cat no. 438748-C1] or RNAscope® 2.5 LS Probe - Mm-Vax1 mRNA – [musculus ventral anterior homeobox 1 (Vax1), cat no. 805108-C1]; RNAscope® 2.5 LS Probe – Mm-Avp-C2 [arginine vasopressin (Avp) mRNA, cat no. 401398-C2]; RNAscope® 2.5 LS Probe – Mm-Vip-C3 [vasoactive intestinal polypeptide (Vip) mRNA, cat no. 415968-C3]; and RNAscope® 2.5 LS Probe – Mm-Nms-C4 [neuromedin S (Nms) transcript variant 1, cat no. 472338-C4]. Stock Mm-Nms-C4 probe was diluted at 1:50 in a pre-diluted C1 probe as recommended by the vendor, whereas stock Mm-Avp-C2 and Mm-Vip-C3 were further diluted to 1:100 in appropriate pre-diluted C1 probe due to saturating signal in the pilot experiment. Tissue slides were counterstained with DAPI and scanned with an Aperio Versa imaging system with 20X objective with customized narrow-width band excitation and emission filter cubes as described (56). The Aperio Cellular IF Algorithm (Leica Biosystems, No: 23CIFWL) was used for automated cell enumeration and segmentation based on nuclear DAPI staining. Cells were classified based on the expression levels of one or more mRNAs. In images taken at P2, *Vax1* staining was oversaturated, so they were re-imaged for proper mRNA visualization. Representative images at P2 were displayed with increased contrast, applied to all channels to compare with P10 and P60 in **Figure 1**.

### GnRH and Kisspeptin Challenges and Hormonal Assays

Hormonal challenges were done using kisspeptin and GnRH intraperitoneal (i.p.) injections at ZT3-4. Kiss-10 (catalog #42-431, Batch 7A, Fisher Science, 30 nmoles/mouse) was injected into males or diestrus females and blood was collected from the mouse from the tail vein before (time 0) and after i.p. injection at time points 5, 10, 15, 30, and 45 minutes. Tail blood was collected before (time 0) and 10 minutes after i.p. injection of GnRH (Millipore Sigma, catalog #L7134, 1 μg/kg dose). For all other serum hormone analyses the mice were killed by cervical dislocation, and blood was collected from the abdominal aorta between ZT3 and ZT6. Blood was allowed to clot for 1 hour at room temperature, then centrifuged (room temperature, 15 minutes, 2,600× g).

Serum was collected and stored at -80°C before analysis for estradiol at the Center for Research in Reproduction, Ligand Assay, and Analysis Core, University of Virginia (Charlottesville), by Luminex analysis for LH and FSH on MILLIPLEX MAP Mouse Pituitary Magnetic Bead Panel (Millipore Sigma #MPTMAG-49k) or a competitive enzyme-linked immunosorbent assay (ELISA) kit (EIACORT, ThermoFisher) for corticosterone. Coefficients of variance (CVs) were based on the variance of samples in the standard curve run in duplicate. Reportable range: estradiol: 3–300 pg/ml, CV = <20%; LH: lower detection limit: 5.6 pg/ml, CV < 15%; FSH: lower detection limit: 25.3 pg/ml, CV < 15%; corticosterone: lower detection limit: 0.87 μg/dl, CV = <20%. Samples were run in singlets.

### *Ex vivo* tissue recordings of PER2::LUC expression

For circadian rhythm organotypic explant studies, tissues from mice expressing the PER2::LUC circadian reporter were collected and analyzed as previously described (57). Male and proestrus and diestrus PER2::LUC females were placed on LD and euthanized at ZT3-4 via isoflurane inhalation and cervical dislocation. The brain was removed immediately and placed in an ice-cold, CO_2_-saturated Hank’s Balanced Salt Solution (HBSS) for approximately 1 hour. Using a Vibratome (Leica), coronal brain sections of 300 μm were collected and the SCN was dissected from the slices in ~2x2 mm squares and placed on a 30 mm Millicell membrane (Millipore-Sigma) in a 35 mm cell culture plate containing 1 mL Neurobasal-A Medium (Gibco) with 1% Glutamax (Gibco), B27 supplement (2%; 12349-015, Gibco), and 1 mM luciferin (BD Biosciences). The lid was sealed to the plate using vacuum grease to ensure an air-tight seal. Plated tissues were loaded into a LumiCycle luminometer (Actimetrics) inside a 35˚C non-humidified incubator at ZT6-6.5, and recordings were started. The bioluminescence was counted for 70 seconds every 10 minutes for 6 days (day 1 – day 7 of recording time). PER2::LUC rhythm data were analyzed using LumiCycle Analysis software (Actimetrics) by an experimenter blind to the experimental group. Data were detrended by subtraction of the 24 h running average, smoothed with a 2 h running average, and fitted to a damped sine wave (LM Fit, damped). The period was defined as the time in hours between the peaks of the fitted curve. Amplitude was defined as the value of the second peak and phase was defined as the time of the first peak. Data from proestrus and diestrus female SCN recordings were pooled as no significant differences in PER2::LUC period or amplitude were found.

### Cell culture and transient transfections

NIH3T3 (American Type Culture Collection) and COS-1 (American Type Culture Collection) cells were cultured in DMEM (Mediatech), containing 10% fetal bovine serum (Gemini Bio), and 1x penicillin-streptomycin (Life Technologies/Invitrogen) in a humidified 5% CO_2_ incubator at 37˚C. For luciferase assays, NIH3T3 cells were seeded into 24-well plates (Nunc) at 30,000 cells per well. For electrophoretic mobility shift assays (EMSA) COS-1 cells were plated at 1.5 million cells/10 cm dish. Transient transfections for luciferase assays were performed using PolyJet™ (SignaGen Laboratories, Rockville, MD), whereas Fugene was used for plasmid overexpression for EMSA, following the manufacturer’s recommendations. Transfection of cells was performed 48 h after the cells were plated. COS-1 cells were transfected with Vax1/DKK-Flag or CMV6/DKK-Flag overexpression plasmids (20 ng/well, Origene Technologies, Rockville, MD) and harvested at sub-confluency 48–56 h after transient transfections in 10 cm dishes (Nunc). Transient transfections for luciferase assays were done following the manufacturer’s recommendations. NIH3T3 cells for luciferase assays were co-transfected with 150 ng/well of Bmal1-luciferase or Per2-luciferase reporters, 100 ng/well thymidine kinase-β-galactosidase reporter plasmid, which served as an internal control (54), as well as mouse Vax1/pCMV6 overexpression plasmid (20 ng/well, Origene Technologies, Rockville, MD), or its empty vector control (pCMV6). To generate the Bmal1-luciferase plasmid the Bmal1 sequence between -966 bp to +140 bp from the *Bmal1* transcriptional start site was excised from the pABpuro-BluF plasmid (Addgene, Plasmid #46824) with PCR primers (F: gggctacaacagaacaactaac, R: taaacaggcacctccgt). The PCR product was inserted into the pGL3-basic backbone between the Mlu-HF and XhoI sites using the Quick Ligation Kit (New England Biolabs). Site directed mutagenesis of the homeodomain binding sites (ATTA and ATTA-like) in the mouse Bmal1-luciferase plasmid was performed using the NEB Q5 Site-Directed Mutagenesis Protocol (New England Biolabs Inc.), following manufacturer’s instructions. Primers for NEB Q5 site-directed mutagenesis were designed using the NEB Base Changer (**Table 1**). To equalize the amount of DNA transfected into cells, we systematically equalized plasmid concentrations by adding the corresponding inactive plasmid backbone. Cells were harvested 24 h after transfection in lysis buffer [100 mM potassium phosphate (pH 7.8) and 0.2% Triton X-100]. Luciferase values were normalized to β-galactosidase values to control for transfection efficiency. Values were further normalized by expression as fold change compared to the pGL3 control plasmid, as indicated in the figure legends. Data represent the mean ± SEM of at least three independent experiments done in duplicate and triplicate.

**Table 1:** Primers used for site-directed mutagenesis in the *Bmal1* regulatory region.

Position	Sequence
-841	TGTCCATAACATGTAATAGAATCTTGCTCA
-796	CTCAGTACTCGCGATTATGCCCCTGCCTCA
-759	CTTGAGGGTTGGAATTACAGACTACGCCAC
-603	AAATGCGCTGGCTATTAGCGCTGTGGTTCC
-537	CACTCTGTGTTCCTAATATGTGGTTTCCTA

Primers used to mutate ATTA sites to GCCG within the Bmal1 promoter plasmid. Position refers to the number of base pairs from the transcription start site. Underlined sequences indicate mutated bases. All primers were designed using NEBase Changer.

### Cytoplasmic and nuclear extracts and Electrophoretic Mobility Shift Assay (EMSA)

COS-1 cells were scraped in hypotonic buffer (20 mM Tris-HCl, pH 7.4, 10 mM NaCl, 1 mM MgCl_2_, 10 mM NaF, 1 mM phenylmethylsulfonyl fluoride, 1x protease inhibitor cocktail; Sigma-Aldrich) and left on ice to swell. Cells were lysed and nuclei were collected by centrifugation (4°C, 1700 g, 4 minutes). Nuclear proteins were extracted on ice for 30 minutes in hypertonic buffer [20 mM HEPES, pH 7.9, 20% glycerol, 420 mM KCl, 2 mM MgCl_2_, 10 mM NaF, 0.1 mM EDTA, 0.1 mM EGTA, 1x protease inhibitor cocktail (Sigma-Aldrich), and 1 mM phenylmethylsulfonylfluoride]. Debris was eliminated by centrifugation (4°C, 20,000 *g*, 10 min), and the supernatant was snap-frozen and stored at -80°C.

Oligonucleotide probes are listed in **Table 1**. All synthetic oligonucleotides were made by IDT (San Diego, CA). Annealed double-stranded oligonucleotides (1 pmol/µl) were end-labeled with T4 Polynucleotide Kinase (New England Biolabs, Ipswich, MA) and [γ^32^P]ATP (7000 Ci/mmol; MP Biomedicals, Solon, OH). Probes were purified using Micro Bio-Spin 6 Chromatography Columns (Bio-Rad). Binding reactions contained 2 µg nuclear protein and 1 fmol of labeled probe in 10 mM HEPES (pH 7.9), 25 mM KCl, 2.5 mM MgCl2, 1% glycerol, 0.1% Nonidet P-40, 0.25 mM EDTA, 0.25% BSA, 1 mM dithiothreitol, and 350 ng poly(dI-dC). For super-shift experiments, 2 µg mouse anti-DKK (Flag antibody, Origene #TA50011) or 2 µg of normal mouse IgG (Santa Cruz Biotechnology, #sc2025) were added to the reaction. Samples were incubated for 20 minutes at room temperature before loading on a 5% non-denaturing polyacrylamide gel in 0.25x Tris-borate EDTA buffer. Gels were run for 2 h at 200 V, dried under vacuum, and exposed to film for 2-5 d at room temperature.

### Statistical analysis

Statistical analyses were performed with GraphPad Prism 8, using Student’s t-test, one-way ANOVA, or two-way ANOVA, followed by *post hoc* analysis by Tukey or Bonferroni as indicated in figure legends, with p *<* 0.05 to indicate significance. All data were analyzed as independent measures except for wheel-running activity, which was analyzed via a two-way repeated-measures ANOVA. PER2::LUC timing of first peak phase relationships was analyzed in R via a Circular Analysis of Variance High Concentration F-Test, with a corrected confidence level of p *<* 0.01667 to account for family-wise error.

## Results

### Characterization of *Vax1* expression in *Vip, Avp*, and *Nms* neurons in the male and female SCN

**Figure 2:**
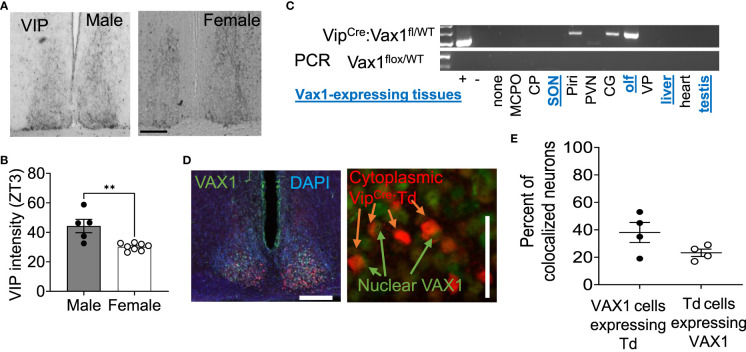
VIP peptide is sexually dimorphic in the SCN. **(A)** Example image and **(B)** quantification of SCN IHC for VIP in adult males and females at ZT3. Scale bar 100µm. Student’s t-test, **, p < 0.01 **(C)** RT-PCR shows Vip^Cre^ recombination of the Vax1^flox^ allele in indicated tissues. Abbreviations stand for olfactory bulb (olf), magnocellular preoptic area (MCPO), caudoputamen (CP), supraoptic nucleus (SON), piraform area (Piri), paraventricular nucleus (PVN), cingulate gyrus (CG), and ventral palidum (VP). Blue, underlined text indicates tissues that are known to express *Vax1*. **(D)** Example images and quantification **(E)** of dual IHC for Vip^Cre^:Td (red, cytoplasm) and VAX1 (green, nuclear) expressing cells in the adult SCN (DAPI, nuclei). Scale 50 µm.

*Vax1* is highly expressed in the developing mouse SCN and becomes refined to the hypothalamus, primarily in the SCN in the early postnatal period (25, 58). Although conditional deletion of *Vax1* in late neuronal development using the Synapsin^Cre^ allele reduced VIP expression in the adult SCN (25), it remains unknown if all VIP expressing neurons co-express VAX1 and how postnatal deletion of *Vax1* in VIP neurons impacts VIP expression. Because VAX1 is highly expressed in the developing SCN, we first asked how VAX1 expression changed after birth and into adulthood in males and females. To answer this, we performed multiplex RNAscope® assay at postnatal day 2 (P2), P10, and P60 (adult) at ZT5 in males and females. We found that all SCN *Vip-*expressing cells at P2 co-express *Vax1* [**Figures 1A, B, G**; male (n = 2) 99.4 ± 0.6%, female (n = 2) 100% ± 0%], a pattern maintained at P10 [**Figures 1C, D, I**; male (n = 4) 99.98 ± 0.021%, female (n = 4) 99.91 ± 0.09%], and P60 [**Figures 1E, F, K**; male (n = 3) 98.80 ± 1.08%, female (n = 4) 100 ± 0%]. In addition to the high co-expression with *Vip*, *Vax1* is highly expressed throughout the SCN at P2, P10, and P60 (**Figures 1A–F**), where both *Avp* and *Nms*-expressing cells also exhibited full overlap with *Vax1* in both sexes (**Figures 1G, I, K**). Interestingly, we found a sex difference in the number of cells expressing *Avp* at P60, where females had fewer *Avp+* cells compared to males [**Figure 1L**; n = 7, t(5) = 5.671, p = 0.0024], a difference that was not present prior to puberty (P10). Although we did not see a significant sex difference in the number of *Vip* neurons at any age, the concentration of *Vip*, as evaluated through *Vip* probe signal intensity, was significantly lower in females than males at P10 [t(3) = 6.01, p = 0.037, not shown] and trended lower at P60 [t(5) = 2.47, p = 0.16, not shown]. To determine if this modest sex difference in *Vip* concentration translated to a sex difference in peptide levels, we performed IHC in adult male and female brains. Adult female mice had a significant reduction in the intensity of VIP peptide in the SCN [**Figures 2A, B**, t(11) = 3.874, p = 0.0026] as compared to males.

As *Vax1* is ~100% co-expressed with *Vip* from P2 until P60 in males and females (**Figure 1**), we next generated a conditional knockout mouse to determine how the loss of VAX1 in VIP neurons would impact SCN function. Using our RNAscope^®^ ISH data from **Figure 1**, we first visually inspected all stained sections for potential *Vax1* expression in non-SCN *Vip* cells. The olfactory bulb is the only additional brain area that expresses *Vax1* which is also targeted by the Vip^Cre^ allele (**Figure 2C**). In the scenario, the Vip^Cre^ allele does target some *Vax1* expressing cells in the olfactory bulb, a change in reproductive behavior could impact fertility data because olfaction is required for normal male reproductive behavior. To validate that the Vip^Cre^ allele targeted VAX1 expressing neurons of the SCN, we generated Vip^Cre^:RosaTdTomato (Vip^Cre^:Td) mice allowing the identification of all neurons that are targeted by the Vip^Cre^ allele (**Figures 2D, E**). Using dual IHC, we quantified the colocalization of VIP- and VAX1-expressing neurons in the SCN. Using tdTomato as a marker for VIP neurons in sections from Vip^Cre^:Td animals, we found 38 ± 7% of SCN tdTomato+ neurons colocalized with VAX1 (n=4, 2 per sex, 36-101 neurons per animal). An average of 23 ± 5% of VAX1 neurons colocalized with tdTomato (n=4, 65-236 neurons per animal). This discrepancy between the RNAscope and dual IHC has numerous potential explanations, as detailed in the discussion.

### Conditional deletion of VAX1 in Vip^Cre^ neurons shortens SCN circadian period in females and males

**Figure 3:**
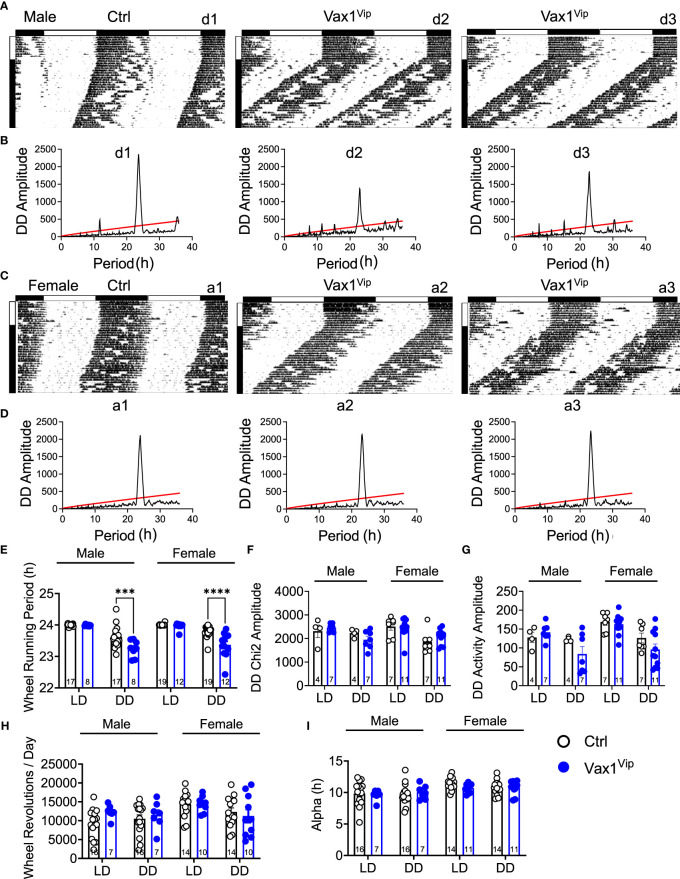
Vax1 deletion within VIP neurons shortens behavioral free-running period in males and females. **(A, B)** Male and **(C, D)** female Ctrl and Vax1^Vip^ mice were single housed with running wheels. **(A, C)** Data show double plotted actogram activity with 14 days in LD12:12 (LD) followed by 28 days in constant darkness (DD). Data are presented in ClockLab normalized format. Horizontal bar above the actograms indicates lights on (white) and lights off (black) during the LD12:12 cycle. **(B, D)** Chi^2^ periodograms during 2 weeks in DD. Matching codes (a1, a2, etc.) on the upper right corner of each actogram and chi^2^ periodogram indicate data from a particular mouse, with variable scaling indicated in the upper left. 14-day average wheel-running data were used for indicated analysis parameters in LD and DD. Average histogram data for **(E)** Wheel-running period, **(F)** Chi^2^ amplitude, **(G)** activity amplitude, **(H)** wheel revolutions, and **(I)** activity duration (Alpha). Number within the bar indicates number of animals in each group. 3-way ANOVA, ***, p < 0.001; ****, p < 0.0001.

**Figure 4:**
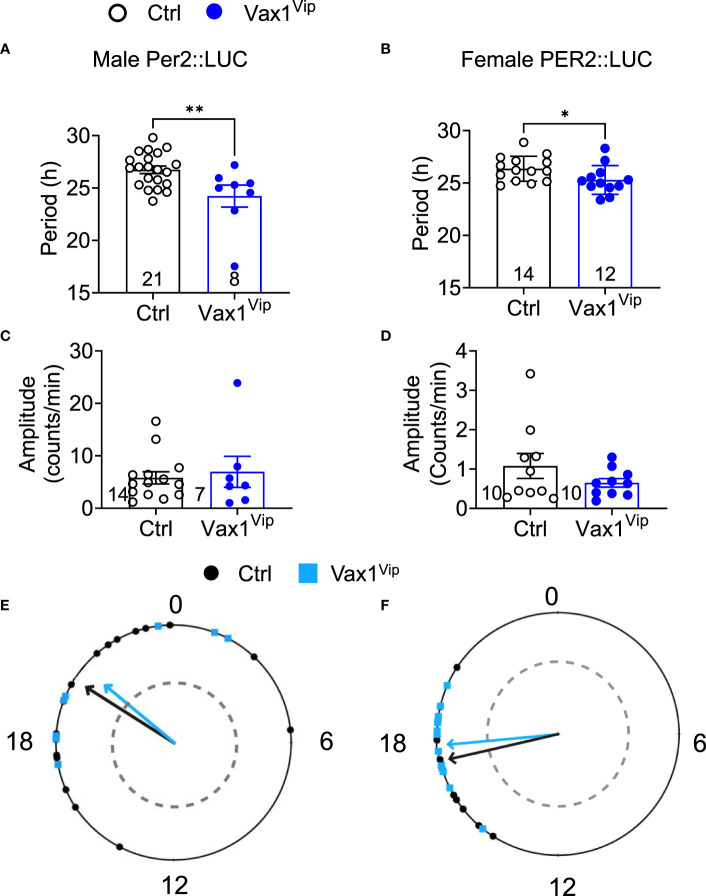
Vax1^Vip^ SCN has a shortened PER2::LUC period in *ex vivo* culture. Histogram of PER2::LUC SCN **(A, B)** circadian period and **(C, D)** amplitude from control and Vax1^Vip^:PER2::LUC females (combined proestrus and diestrus) and males. Statistical analysis by Student’s t-test, *, p<0.05 **, p < 0.01, n = 7-16. **(E)** PER2::LUC phase (time of first peak) in the SCN of control and Vax1^Vip^:PER2::LUC males and **(F)** females, n = 7-16. Mean times of first peak are indicated by vector lines, and symbols indicate individual data points. Data were analyzed via the Rayleigh Test of Uniformity, where crossing the dotted gray line indicates significant clustering (p < 0.05), and the Watson’s Two-Sample Test of Homogeneity. No significant differences were found in females between estrous stages.

To determine the role of VAX1 in VIP neuron circadian output, we evaluated the wheel running behavior of Ctrl and Vax1^flox/flox^:Vip^Cre^ (Vax1^Vip^) mice in LD and constant darkness (DD). Wheel running patterns in LD of Ctrl and Vax1^Vip^ females and males were comparable (**Figures 3A–I**, LD). Light is a strong entraining signal of the SCN, and activity rhythms in LD can mask weakened SCN function (59). Following the initial LD period, mice were placed in DD for 28d to assess the endogenous free-running period. Both male and female Vax1^Vip^ mice showed a significantly shortened free-running period (Tau) compared to Ctrls (**Figure 3E**, two-way ANOVA, male p = 0.0004, female p = <0.0001) with no differences in Chi^2^ amplitude (Qp). There were no changes in the number of wheel revolutions per day or activity duration (alpha, **Figures 3F–I**). To determine if the shortening of the free-running period during DD resulted from a change in the endogenous SCN circadian period, we generated triple transgenic mice crossing Vax1^Vip^ mice with the PER2::LUC reporter mouse (60). In agreement with the significantly shortened behavioral period of Vax1^Vip^ males and females on DD, we found that the SCN of Vax1^Vip^:PER2::LUC mice showed a significant shortening in period as compared to Ctrl males [**Figure 4A**, t(27) = 2.936, p = 0.0067] and females [**Figure 4B**, t(24) = 2.125, p = 0.0440]. No differences were found in the amplitude or phase relationships of PER2::LUC in the SCN of Vax1^Vip^ males or females as compared to Ctrl, indicating that the rhythms in the SCN are not misaligned or significantly weakened (**Figures 4C–F**). Together these data show that loss of VAX1 in VIP neurons shortens SCN circadian output in both males and females.

### VAX1 regulates molecular clock gene expression in VIP neurons through a direct mechanism

**Figure 5:**
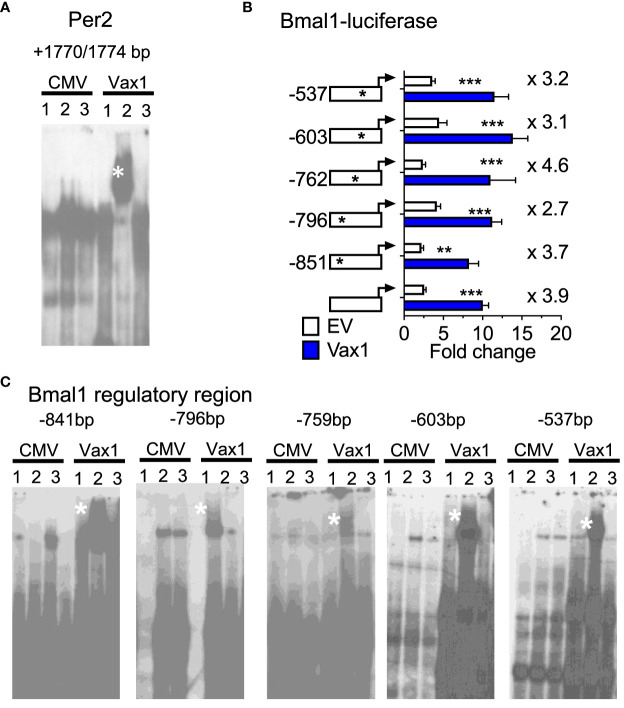
VAX1 binds directly to regulatory regions of molecular clock genes *Per2* and *Bmal1.* **(A, C)** EMSA assay of COS-1 cells, represents in Lane 1: pCMV-Flag (CMV, empty vector), Lane 2: Vax1-Flag plasmid and Lane 3: Vax1-Flag plasmid + anti Flag antibody. White star indicates super shift. Example gels of n = 3. **(B)** Transient transfections of NIH3T3 cells with the mouse *Bmal1* regulatory region driving luciferase (Bmal1-luciferase) with and without Vax1 overexpression vector (20 ng) or its empty vector (EV, pCMV6, 20 ng). Numbers indicated with the stars on the regulatory regions refer to ATTA sites that have been mutated (see **Table 1**). Statistical analysis by Two-way ANOVA mixed effect model, *, p < 0.05; **, p < 0.01; ***, p < 0.001, n = 4-6 in duplicate or triplicate.

**Figure 6:**
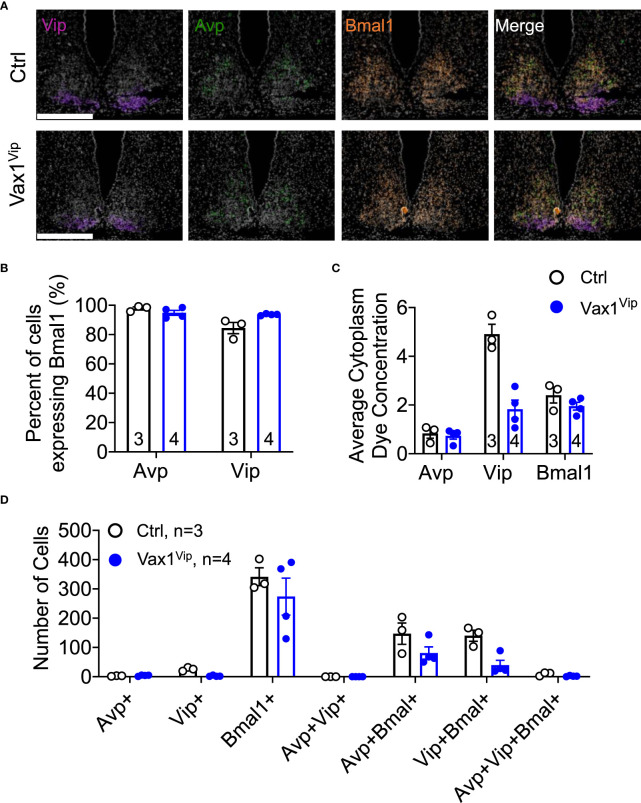
Loss of Vax1 in VIP neurons does not reduce *Vip* and *Bmal1* expression in the SCN. RNAscope® assay at ZT16 in the SCN of proestrus Ctrl and Vax1^Vip^ females. **(A)** Example images of RNAscope® assay for *Vip* (blue), *Avp* (red) and *Bmal1* (green). N = 3-4 per group. Scale bar 300 µm. **(B)** Percentage of cells that co-express *Bmal1* with *Avp* or *Vip*. Mann-Whitney, p > 0.05. **(C)** Average cytoplasm dye concentration reflecting mRNA transcripts for *Avp*, *Vip*, and *Bmal1* Mann-Whitney, p > 0.05. **(D)** Number of cells expressing indicated combinations of *Avp*, *Vip*, and *Bmal1*. Mann-Whitney, p > 0.05.

A shortened SCN period can be driven by both changes in SCN peptide expression and changes in molecular clock gene expression. We have previously shown that VAX1 promotes Per2-luciferase plasmid expression using transient transfection assays (25). However, we have not yet demonstrated whether this action is direct or indirect. To assess if VAX1 directly binds with our top candidate ATTA site of the mouse *Per2* DNA regulatory region (25), we used EMSA. We found that VAX1 directly binds the ATTA site at +1774/1770bp from the transcriptional start site of the mouse *Per2* gene (**Figure 5A**, Super shift is indicated by *). In addition to ATTA sites in the *Per2* regulatory region, the *Bmal1* regulatory region also contains numerous ATTA sites. Using transient transfections, we found that VAX1 promotes *Bmal1-*luciferase expression (**Figure 5B**). Site-directed mutagenesis of ATTA-like sites in the *Bmal1* regulatory region (**Table 1**) showed a modest increase in fold-change of VAX1-driven *Bmal1-*luciferase expression in transfected cells (**Figure 5B**). Interestingly, VAX1 can directly bind to all the identified ATTA sites tested by EMSA (EMSA, **Figure 5C**, supershift indicated by *). This identifies for the first time that VAX1 can directly bind to the regulatory regions of *Per2* and *Bmal1* and provides a mechanism by which changes in VAX1 expression can directly impact molecular clock function. Next, to determine if loss of VAX1 in VIP neurons would significantly impact *Bmal1* expression in *Vip* neurons, we performed RNAscope^®^ for *Bmal1, Vip*, and *Avp* in the adult SCN of Ctrl and Vax1^Vip^ females (**Figure 6**). Note these experiments were completed in proestrus at ZT16, with the goal of having the most hormonally challenging environment in the female body present at the time of sample collection. We found that in Ctrl and Vax1^Vip^ females almost 100% of *Vip* and *Avp* neurons co-expressed *Bmal1* (**Figures 6A, B**). Despite a trend in a reduction in *Vip* in the SCN of Vax1^Vip^ females (**Figure 6C**), as well as a trend in the reduction in cells co-expressing *Vip* and *Bmal1* (**Figure 6D**), no significant difference in any of the studied transcripts, or colocalization of transcripts were identified (**Figures 6C, D**).

### Vax1^Vip^ females have lengthened estrous cycles and deregulated sex steroids, whereas males have increased sperm count

**Figure 7:**
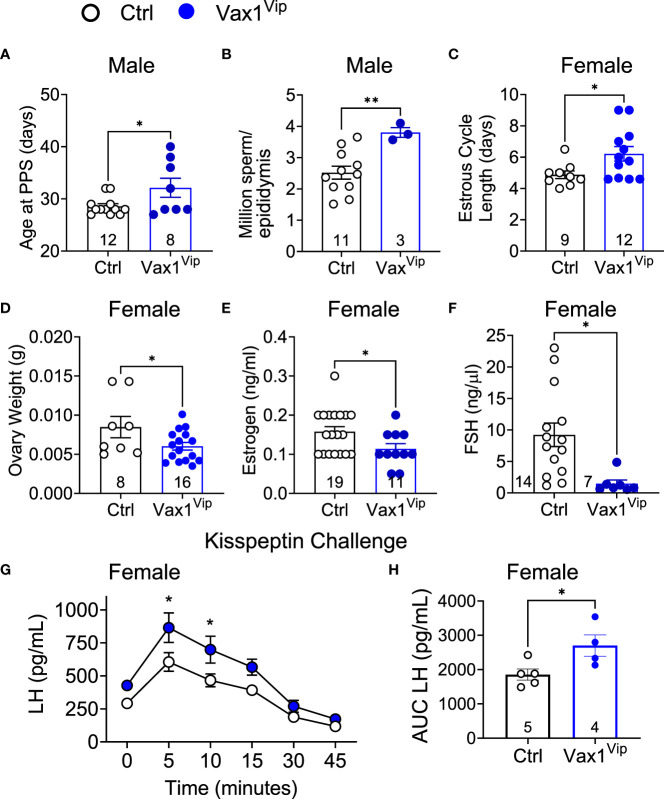
Vax1^Vip^ females have a reduction in ovary weight, estrogen, and FSH, as well as an increased sensitivity to kisspeptin. **(A)** Age at preputial separation (PPS) and **(B)** million sperm per epididymis in Ctrl and Vax1^Vip^ males, n indicated in graphs, Student’s t-test, *, p<0.05; **, p <0.01. **(C)** Estrous cycles were evaluated in females, and average estrous cycle length was established. Student’s t-test, *, p < 0.05. **(D)** Ovary weight, **(E)** circulating estrogen and **(F)** circulating FSH of diestrus females. n indicated in graphs, Student’s t-test, *, p < 0.05. **(G)** Circulating LH levels in diestrus females evaluated over a 45-minute time period in response to an i.p. kisspeptin injection. Mixed Effects analysis, *, p < 0.05. and **(H)** the resulting area under the curve. Student’s t-test, *, p < 0.05.

The SCN provides daily neuronal and hormonal signals aligning circadian timekeeping in peripheral reproductive tissues allowing coordination between hormone release and increased tissue sensitivity improving reproductive function (25, 27, 35, 57). To determine if Vax1^Vip^ mice have misaligned circadian phase of their reproductive tissues, we recorded PER2::LUC expression in the pituitary, ovary (female), uterus (female), and epididymis (male) of triple transgenic mice. No tissues were found to have significant differences in period (**Table 2**), amplitude (**Table 2**), or time of first PER2::LUC peak (**Table 2**, phase). These data indicate that the weakened SCN output of Vax1^Vip^ mice does not significantly impact circadian timekeeping in the studied peripheral tissues but does not preclude disruptions to fertility. To determine if reproductive function is impacted in Vax1^Vip^ mice, we first evaluated pubertal onset. At pubertal onset, body weight was comparable between Ctrls and Vax1^Vip^ in both sexes (**Table 3**). Male pubertal onset, as evaluated by preputial separation (PPS) was slightly delayed [**Figure 7A**, t(18) = 2.211, p = 0.040], whereas female pubertal onset, as assessed through vaginal opening (VO) and first estrous, were comparable between Ctrls and Vax1^Vip^ females (**Table 3**). There was no impact on male reproductive function (**Table 3**) apart from significantly increased total sperm count [**Figure 7B**, t(12) = 3.101, p = 0.009]. Vax1^Vip^ males had normal testis size and percent motile sperm (**Table 3**). The increase in total sperm pool was not associated with changes in basal LH and FSH levels in males (**Table 3**). Both Vax1^Vip^ males and females were comparable to Ctrls for the number of litters generated in 90 days, days to first litter, and litter sizes (**Table 3**). Despite the normal fertility in females, Vax1^Vip^ females had a significant lengthening of the estrous cycle [**Figure 7C**, t(19) = 2.307, p = 0.033] with a similar amount of time spent in each cycle stage as compared to Ctrls [Two-way ANOVA, F (1, 10) = 2.500, P=0.1449]. This lengthening in estrous cycles was associated with a reduction in FSH, estrogen, and ovarian weight in Vax1^Vip^ females (**Figures 7D–F**), but did not impact basal LH or uterine weight in diestrus (**Table 3**).

**Table 2:** Summarized data of male and female Vax1^Vip^:PER2::LUC recordings.

	Ctrl (Avg ± SEM)	Vax1^Vip^ (Avg ± SEM)	Student’s t-test, P
Ovary Period (h)	25.6 ± 0.3	25.2 ± 1.5	n = 3-6, P = 0.87
Ovary Amplitude (counts/min)	27.4 ± 11.2	8.7 ± 2.6	n = 3-6, P = 0.40
Uterus Period (h)	25.7 ± 0.5	27.2 ± 1.7	n = 3-6, P = 0.25
Uterus Amplitude (counts/min)	43.3 ± 9.6	9.1 ± 7.2	n = 3-6, P = 0.10
Female Pituitary Period (h)	24.2 ± 0.2	25.5 ± 0.3	n = 3-6, P = 0.18
Female Pituitary Amplitude (counts/min)	10.5 ± 3.3	15.4 ± 11.5	n = 3-5, P = 0.58
Female Arcuate Period (h)	25.3 ± 0.7	24.1 ± 0.6	n = 3-5, P = 0.39
Female Arcuate Amplitude (counts/min)	1.1 ± 0.4	1.3 ± 0.2	n = 3-5, P = 0.77
Epididymis Period (h)	24.5 ± 0.2	25.5 ± 0.5	n = 8-9, P = 0.12
Epididymis Amplitude (counts/min)	12.9 ± 4.9	12.7 ± 1.0	n = 8, P = 0.98
Epididymis Phase (h)	4.5 ± 0.1	4.1 ± 0.1	n = 8-9, P = 0.81
Male Pituitary Period (h)	25.3 ± 0.3	24.9 ± 0.6	n = 8-23, P = 0.447
Male Pituitary Amplitude (counts/min)	16.5 ± 2.5	9.8 ± 2.6	n = 8-20, P = 0.14
Male Arcuate Period (h)	24.7 ± 0.5	24.9 ± 0.4	n = 8-22, P = 0.84
Male Arcuate Amplitude (counts/min)	1.9 ± 0.4	1.8 ± 0.6	n = 8-22, P = 0.88

PER2::LUC lumicycle data from male and proestrus female tissues. Phase data were analyzed using a Rayleigh test followed by a Watson two-sample test of homogeneity.

**Table 3:** Fertility parameters in Vax1^Vip^ mice.

	Ctrl (Avg ± SEM)	Vax1^Vip^ (Avg ± SEM)	Student’s t-test, P
Age at VO (days)	28.7 ± 0.5	29.1 ± 0.9	n = 10-26, P = 0.67
Weight at VO (g)	12.0 ± 0.2	12.5 ± 0.5	n = 10-26, P = 0.36
Weight at PPS (g)	13.1 ± 0.4	14.0 ± 0.7	n = 8-12, P = 0.22
Diestrus uterus weight (mg)	71.6 ± 9.5	71.5 ± 7.5	n = 8-16, P = 0.99
Age at first estrus (days)	34.7 ± 0.9	34.0 ± 1.4	n = 7-22, P = 0.70
Testis weight (mg)	103.8 ± 4.4	100.4 ± 3.5	n = 4-6, P = 0.59
LH (ng/ml) diestrus female	0.51 ± 0.13	0.17 ± 0.04	n = 7-14, P = 0.09
LH (ng/ml) male	0.35 ± 0.08	0.40 ± 0.19	n = 10-14, P = 0.83
FSH (ng/ml) male	12.58 ± 1.13	11.41 ± 1.12	n = 12-14, P = 0.47
Percent Motile Sperm	35.09 ± 2.41	31.21 ± 4.58	n = 3-9, P = 0.70
Female Litter Size	7.14 ± 1.55	8.34 ± 0.84	n= 6-22, P = 0.16
Male Litter Size	7.14 ± 1.55	7.48 ± 1.52	n= 10-22, P = 0.79
Female Litters in 90 days	2.05 ± 0.65	2.33 ± 0.82	n= 6-22, P =0.61
Male Litters in 90 days	2.05 ± 0.65	2.43 ± 0.79	n= 7-22, P = 0.38
Female Days to first litter	25.69 ± 7.86	21.33 ± 1.51	n= 6-16, P = 0.31
Males Days to first litter	25.69 ± 7.86	26.64 ± 6.05	n= 11-16, P = 0.91

Pubertal onset was evaluated by vaginal opening (VO) in females and preputial separation (PPS) in males. Gonadal, uterine, and circulating hormone values are from adult Vax1^Vip^ males and diestrus/metestrus females. Statistical analysis by Student’s t-test.

### Reduced VIP in the SCN of Vax1^Vip^ mice is associated with increased GnRH neuron sensitivity to kisspeptin in females, but not in males

VIP neurons from the SCN project directly to GnRH neurons and indirectly through AVP to kisspeptin neurons. The anterior pituitary releases LH into the circulation upon GnRH release at the median eminence, allowing an indirect approach to study GnRH neuron function. To determine if the reduction in VIP in the Vax1^Vip^ SCN impacted GnRH neuron response to kisspeptin, we performed hormone challenges in mice. We first confirmed that the pituitary responded to GnRH by increasing LH release through an i.p. injection of GnRH. As expected, the fold change in LH in response to a GnRH challenge was comparable between Ctrl (9.07 ± 1.97, n = 4) and Vax1^Vip^ males [12.09 ± 2.43, n = 6, t(8) = 0.885, p= 0.401], and between Ctrl (10.50 ± 3.37, n=8), and Vax1^Vip^ females [6.95 ± 3.84, n = 4, t(10) = 0.641, p= 0.535]. To assess if the GnRH neuron response to kisspeptin was impacted in Vax1^Vip^ males and females, we next performed an i.p. kisspeptin challenge. There were no differences between LH release in Vax1^Vip^ (5798 pg/mL ± 583, n = 4) males and Ctrls [5325 pg/mL ± 670, n = 5, t(7) = 1.11, p = 0.303]. In contrast, Vax1^Vip^ females had an increased release of LH in response to kisspeptin at 5 and 10 minutes as compared to Ctrls (**Figure 7G**, mixed-effects analysis, 5 minutes p = 0.0102, 10 minutes p = 0.0257), as well as an overall increase in LH release [**Figure 7H**, t(7) = 2.560, p = 0.0376]. Such alteration in the neuroendocrine network regulating LH release would be expected to impact female estrous cyclicity, which relies on precisely timed hormone release and sex-steroid-feedback.

### Hypothalamic imbalance between *Vip* and *Avp* in the Vax1^Vip^ hypothalamus is associated with increased basal corticosterone and depressive-like symptoms in females

**Figure 8:**
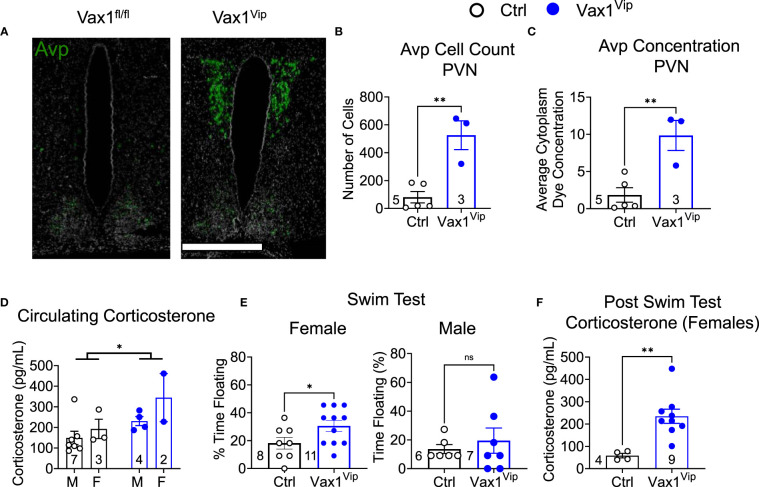
Vax1^Vip^ females have increased *Avp* in the PVN, increased corticosterone, and increased depressive-like behaviors. **(A)** Example images of RNAscope® detection for *Avp* in the SCN and paraventricular nucleus (PVN) of proestrus females at ZT16. Scale bar is 600 µm, n=3-5. Quantification of RNAscope® assay by **(B)** cell counts and **(C)** dye concentration of *Avp* in the PVN, n=3-5, Student’s t-test, **, p <0.01. **(D)** Corticosterone was measured at ZT3-5 in males (M) and females (F), Two-way ANOVA, *, p<0.05. **(E)** To test depressive-like behavior, metestrus female or male mice were tested at ZT13 by a Porsolt forced swim test, and the % time floating assessed. N indicated in graph, Student’s t-test, *, p<0.05. **(F)** Corticosterone levels in circulating blood 1 h following the Porsult forced swim test. n = 4-9, Student’s t-test, **, p<0.01.

AVP is expressed outside the SCN and is highly expressed in the paraventricular nucleus (PVN), a direct target of SCN neurons. The PVN is a well-established relay site playing a significant role in several autonomic functions, including stress (61–63). To determine how *Avp* levels were impacted in the hypothalamus of Vax1^Vip^ females, we analyzed *Avp* using RNAscope^®^ assay. Interestingly, we found that Vax1^Vip^ females, which have comparable *Avp* mRNA in the SCN to Ctrl (**Figures 6C, D**), display a significant increase in *Avp* transcript and cell numbers in the PVN (**Figures 8A–C**). This increase of *Avp* in the PVN of Vax1^Vip^ females provides a potential link to the activation of the stress axis. In agreement with this, basal corticosterone levels were overall increased at ZT3 in Vax1^Vip^ mice [**Figure 8D**, two-way ANOVA, F(1,12) = 5.868, p = 0.032]. Finally, to determine if these known risk factors for depression would reflect an increase in depressive-like behavior in Vax1^Vip^ mice, we tested males and females in the Porsolt forced swim test. We found that Vax1^Vip^ females (metestrus, ZT13) exhibited increased depressive-like behaviors as shown by an increase in the percentage of time floating (**Figure 8E**) as compared to Ctrl females [t(17) = 2.121, p = 0.0489], while Vax1^Vip^ males were comparable to Ctrls [t(11) = 0.5889, p = 0.5678]. The increased time floating in the Porsolt forced swim tests of the Vax^Vip^ females correlated with increased circulating corticosterone 1 h after the swim test [**Figure 8F**, t(11) = 3.595, p = 0.0042].

## Discussion

This work explores VIP neurons within the SCN as a neurological point of overlap between circadian disruption, reproduction, and depressive-like behaviors. Here, we leverage the highly localized co-expression between the homeodomain transcription factor VAX1 in VIP neurons of the SCN to develop a conditional knockout mouse model that exhibits abnormal circadian timekeeping, reproductive axis function, and mood. Excitingly, this novel mouse model suggests that VIP neurons might provide a shared neurological underpinning between reproductive and mood disorders.

### *Vax1* is co-expressed in *Vip, Nms*, and *Avp* neurons from development to adulthood and regulates molecular clock gene expression

The SCN retains a high expression of numerous homeodomain transcription factors after development (26, 46, 64–66), including *Vax1*. *Vax1* is critical in brain and neuronal development (25, 46, 47, 53, 67) and highly expressed in the developing mouse brain before it becomes refined to the adult hypothalamus, primarily in the SCN, during the early postnatal period (25, 58). We have previously shown that conditional deletion of *Vax1* in late neuronal development using the Synapsin^Cre^ allele reduced VIP expression in the adult SCN (25), but this previous study did not address how many VIP neurons co-expressed *Vax1*. Here we find that ~100% of *Vip*-expressing neurons co-express *Vax1* at P2 and maintain a close to 100% co-expression at P10 and P60. In addition, we show that *Vax1* is also co-expressed by ~100% of *Nms* and *Avp* neurons at P2, P10, and P60. This shows for the first time that *Vax1* is highly expressed in three primary SCN neuron populations from the early postnatal period into adulthood, suggesting a role of VAX1 in regulating the function of these neuronal populations. The close to 100% co-expression of *Vax1* and *Vip* strengthens the value of our conditional knock-out model as a novel tool to specifically study SCN VIP neurons. This high level of overlap, coupled with the primary restriction of *Vax1* expression within VIP neurons in adulthood to the SCN (25, 58), allows for targeted impairment of VIP neurons within the SCN alone. This approach allows us to build upon previous work that identified the importance of VIP in circadian and reproductive function using VIP knockout mice (36), while avoiding some pitfalls of other methods of selectively targeting VIP neurons within the SCN, such as damage to other neurons or brain nuclei via surgical methods due to the ventral location of the SCN (9, 68). One concern with conditional knockout mice is that off-target recombination may impact other brain or peripheral functions or have a negative impact on development. In our model, we found Vip^Cre^-mediated recombination outside of the SCN was restricted to the olfactory bulb, which also expresses *Vax1*. However, as a functioning olfactory bulb is pivotal to male mating (69) and our male mice bred normally, it is likely that the olfactory bulb is not significantly affected in this model. We also generated Vip^Cre^ : Td mice to identify neurons that were targeted by the Vip^Cre^ allele prior to tissue collection to validate that the Vip^Cre^ allele specifically targeted VAX1 expressing neurons of the SCN. We confirmed that SCN tdTomato+ neurons colocalized with VAX1, and VAX1 neurons colocalized with tdTomato, although these percentages were lower than predicted by RNAScope^®^ ISH assay. The differences in co-localization between mRNA and protein results could reflect different sensitivity limits between the visualization approaches, or alternatively may provide evidence that not all *Vax1* mRNA is being translated into protein. Another possibility is that VAX1 expression could be circadian at the mRNA and/or protein level, leading to differences in expression that are time-of-day dependent, although future studies will be needed to determine this.

As a transcription factor that binds to ATTA and ATTA-like sites, a common sequence in the DNA, VAX1 has a high number of genes it can potentially regulate. Here, we build upon our previous work that determined the ability of VAX1 to promote Per2-luciferase expression (25) by providing evidence that this occurs via direct binding of VAX1 to regulatory regions of *Per2*. In addition to *Per2*, another core component of the molecular clock, *Bmal1* also contains numerous ATTA-like sites in its regulatory region. We found here that VAX1 can directly bind to all the identified *Bmal1* ATTA sites tested by EMSA, in addition to promoting Bmal1*-*luciferase expression. This work provides a mechanism by which changes in VAX1 expression can directly impact molecular clock function by VAX1 binding to the regulatory regions of *Per2* and *Bmal1*. Excitingly, Vax1^Vip^ mice presented with a shortening in SCN PER2::LUC period in *ex vivo* recordings, as well a shortened free-running period, together supporting a role of VAX1 as a novel regulator of both molecular clock expression and function. Future work will be required to determine if a loss of VAX1 in VIP neurons causes a circadian phase shift in VIP neurons, and/or if a loss of VAX1 leads to a reduction in molecular clock transcript expression, which could impact clock-controlled gene expression and phase.

### Circadian timekeeping is impaired in Vax1^Vip^ mice, where differences in SCN peptide transcript levels may underlie sex-specific vulnerability to circadian disruption

VIP neurons within the SCN are an important coordinator of the circadian timekeeping system (37). Given this, it is no surprise that the Vax1^Vip^ mouse model demonstrates altered SCN output, as indicated by the shortened Vax1^Vip^ free-running period in both sexes. This finding is consistent with work done by others indicating that a decrease in VIP within the SCN results in a shortened free-running period (36). However, VIP rarely acts alone in the regulation of circadian behaviors, and other peptides and components of the molecular clock exert strong influences on locomotor period (70). A shortened free-running period can be caused by an increase in SCN AVP (71–73) or reductions in *Bmal1* expression (74). Others have found that *Bmal1* is expressed rhythmically in *Avp*- and *Vip*-expressing neurons (75) and that deletion of *Bmal1* from *Avp*-expressing neurons can lengthen free-running periods in mice (76). As Vax1^Vip^ females do not show significant changes in *Avp* in the SCN, and only trended towards decreased *Vip* expression, in addition to a non-significant reduction in neurons co-expressing *Bmal1* and *Vip*, the cause of the shortened period in Vax1^Vip^ mice remains unknown. Future work will aim at determining if these trending reductions in *Vip*, combined with a trend in reduced *Bmal1* expression in *Vip* neurons together might contribute to the shortened SCN period of Vax1^Vip^ mice.

Sex differences are well-documented in SCN morphology and cellular function, previously reviewed in several studies (77, 78). There is strong evidence that VIP is sexually dimorphic, with increased VIP expression in human males (79, 80), as well as increased *Vip* transcript in male nocturnal laboratory rats (81) and diurnal Nile Grass rats (82). Our data support and extend these findings, where Ctrl male mice also exhibit increased *Vip* transcript and VIP compared to females. A potential mechanism guiding this sex difference could rely on the influence of the gonadal hormones estradiol and testosterone, which modulate VIP expression in the SCN (81–84). Our data, and others, suggest that sex differences in both *Vip* transcript and peptide levels (78) occur post-puberty. Recent work has demonstrated detailed spatial patterning of the onset and development of *Avp* and *Vip* transcription (85). Our data support the conclusion that SCN *Vip* neuron development is not complete at P10; however, we find decreased *Vip* transcript and protein in the adult female SCN compared to males, while increased VIP-TdT+ cell numbers were found in a lateral cluster of the SCN (85). There are several potential rationales for these differences, including delays between transcription onset and Vip^Cre^-driven TdTomato expression that may lead to different results from our mRNA transcript measure. Nevertheless, these neuropeptide sex differences highlight the need for continued investigation in both males and females to further our understanding of how SCN function drives circadian output in both sexes and the role of sex steroids therein.

### Vax1^Vip^ males exhibit normal fertility, while females display modest dysregulation of the reproductive axis

Circadian timekeeping is essential for coordinating hormone release and increased tissue sensitivity within reproductive tissues (25, 27, 35). The SCN modulates the timing of hormone release through direct and indirect projections to GnRH neurons, which in turn regulate the release of LH and FSH (86–88). These hormones are required for reproductive health through the production of testosterone and spermiogenesis in males and ovulation, embryo implantation, and follicular health in females (89–91). Though more frequently studied in females, current studies suggest that severe circadian disruption, through changes either in light exposure or via direct disruption to the SCN, may have a mild influence on male fertility (92). Interestingly, we found that Vax1^Vip^ males had delayed pubertal onset. Given the importance of both SCN *Avp* and *Vip* in LH release (32, 93, 94), a required hormone for pubertal onset (95), it will be of interest for future studies to examine SCN neuropeptide expression in Vax1^Vip^ males undergoing puberty. Interestingly, aside from delayed pubertal onset, there were no significant deficits in Vax1^Vip^ male reproductive function, and Vax1^Vip^ males displayed an increase in total sperm through an unknown mechanism. These data support a theory that a less stringent circadian control may favor male fertility. Together our data, coupled with evidence from genetic knockout and light-disruption studies (92, 96, 97), indicate that male fertility is resilient when faced with circadian challenges.

In contrast to males, circadian disruption and impaired SCN function are known to have a strong impact on female reproduction (27, 98–100). VIP, in particular, has an important role in the female reproductive axis, where VIP knockout females have lengthened estrous cycles and are sub-fertile, resulting in fewer liters of smaller sizes (19). It is likely that some of the sub-fertility in full body VIP knockout females is driven by VIP neurons within the SCN, as our Vax1^Vip^ females also displayed lengthened estrous cycles. Our data are further supported by work in progress, showing that surgically ablated VIP neurons within the SCN also lengthened estrous cycles (101). Although Vax1^Vip^ estrous cycles were lengthened, we did not see changes in litter sizes or number of litters that are associated with full VIP knockout females. Taken together, these data indicate estrous cycle length is influenced by Vip-expressing neurons within the SCN.

The estrous cycle is regulated by hormonal feedback throughout the reproductive axis. Within the hypothalamus, VIP neurons directly project onto GnRH neurons to regulate the frequency of GnRH release to the pituitary (31) and indirectly through projections onto AVP neurons in the anteroventral paraventricular nucleus to regulate kisspeptin neurons that modulate the surge release of GnRH needed for ovulation (102, 103). Although only a single dose of kisspeptin was tested, we found that Vax1^Vip^ females had a greater release of LH in response to a kisspeptin challenge than Ctrls, a difference we did not observe in the males. The normal pituitary response to GnRH and normal circadian rhythms in *ex vivo* pituitary explants of Vax1^Vip^ females, combined with the absence of *Vax1* in gonadotropes as shown by qPCR in isolated gonadotrope cells from female mice (53, 104) and single cell RNAseq [personal communication, (104, 105)], suggest it is unlikely that the increased LH release in response to kisspeptin is associated with abnormal gonadotrope function. One possibility for the sex difference in kisspeptin-induced changes in LH release in Vax1^Vip^ mice might be linked to differences in the neuronal circuit encompassing the sexually dimorphic anteroventral periventricular nucleus kisspeptin neurons (106). This neuronal population is larger in females than in males and plays a central role in the LH surge (107, 108). Interestingly, a comparable increased sensitivity to a kisspeptin challenge in females was also observed in full body *Bmal1* knockout mice (8), where the mechanism for this increase remains unknown. Future work to determine how neuronal network changes, with or without intact molecular clock function, impact GnRH neuron sensitivity to kisspeptin will be of interest. Although the kisspeptin challenge elicited a greater LH release in the Vax1^Vip^ females than Ctrls, basal LH levels were comparable to Ctrls, and Vax1^Vip^ females exhibited a decrease in circulating FSH. LH and FSH production and release are controlled in a great part by the pulsatile pattern of GnRH release (87, 109, 110). Repeated blood sampling of Vax1^Vip^ mice would have been an ideal approach to assess for changes in pulsatile hormone release, however, as Vax1^Vip^ mice present with normal litter sizes, time to first litter, ovarian phase, which indicate overall normal ovarian function, and an increased activation of the stress axis, which is a suppressor of GnRH release (111), we decided against completing a LH and FSH pulse analysis due to the confounding effect of corticosterone on these data. In the future, we hope to better elucidate the relationship between stress and pulsatile hormones in this mouse model.

### VAX1 in postnatal VIP neurons regulates female depressive-like behavior

Changes in the neuroendocrine regulation of FSH release from the pituitary in Vax1^Vip^ mice is a potential pathway causing the reduction in estrogen of these mice. FSH is a limiting factor in the conversion of testosterone into estrogen in the granulosa cells of the ovary (112, 113), from where estrogen enters the general circulation. It is important to note that both FSH and estrogen are circadian (114, 115), thus a limit of this study, with blood sampling at a single time point, is our inability to assess if the reduction in these hormones might be due to a phase shift in hormone release. While primarily associated with its role in reproduction, estrogen has a multitude of functions, including altering the sensitivity of neuronal circuits (116, 117), regulating the activity of the stress axis (118), and displaying strong correlations with mood (119–121). Low estrogen has been correlated with depressive-like behaviors in women (122). In rodents, increasing estrogen has been shown to exert an anti-depressant-like effect during the Porsolt forced swim test, a test that is thought to reflect depressive-like behavior in rodents (123, 124). Additionally, an imbalance of progesterone and estrogen is associated with a higher incidence of mood disorders, including premenstrual dysphoric disorder (PMDD), a depressive disorder that presents with severe physical and physiological symptoms during the luteal phase of the menstrual cycle (125–128). In addition to recapitulating the low estradiol (or imbalance of progesterone and estrogen) as a risk factor for mood disorders, Vax1^Vip^ females also display another hallmark of PMDD, weakened SCN output (129, 130). Excitingly, Vax1^Vip^ females (but not males) have increased depressive-like behavior. Taken together, these data point to a novel role of VAX1 in regulating VIP neuron modulation of mood and the reproductive axis and raise the potential of Vax1^Vip^ females to serve as a new model for mood disorders that are tied to reproductive cycles, such as PMDD. Furthermore, VIP-and AVP-expressing neurons contribute to the regulation of the stress axis (131–134). Stressful situations result in an increase in signal from the hypothalamus, which translates to higher levels of corticosterone via activation of the hypothalamic-pituitary-adrenal (stress) axis. Notably, the hypothalamus is comprised of several nuclei, including the SCN (135) and the PVN (136, 137), with varying roles and contributions to the stress axis. Within the SCN, reductions in *Vip* have been correlated with increased stress (138), whereas the VIP neuron target, the PVN, is a central relay station of the stress axis (63, 139). Thus, the reduction of *Vip* in the SCN of Vax1^Vip^ females is a likely contributing factor in the increased corticosterone levels found in Vax1^Vip^ females, both at baseline and in response to a stressor. Specifically within the PVN, AVP is known to stimulate the stress axis (140, 141), and AVP expression in the PVN is comparable between control and VIP knockout males (142). This suggests that the reduction in VIP in the SCN of Vax1^Vip^ mice may not be driving the changes in *Avp* mRNA within the PVN, but more likely is the result of other VAX1 targets that impact VIP neuron communication with PVN neurons, such as GABA (143, 144).

## Conclusion and summary

Due to the abundance of VIP throughout the brain and body, it is difficult to study subsets of neurons expressing VIP without invasive surgery, which can lead to damaged brain tissue and a variety of other complications. In this study, we leveraged the close to 100% overlap of *Vax1* expression specifically within SCN *Vip* neurons, to generate a conditional knockout mouse model to study this subset of VIP neurons. We found that deletion of *Vax1* from SCN VIP neurons results in mice with altered circadian rhythms. Excitingly, Vax1^Vip^ females had disrupted reproductive axis function, low estrogen, and high corticosterone, as well as an increase in depressive-like behaviors. Together, these data provide us with an exciting new model to study the genetic and neurological overlap between circadian disruption, female reproductive health, and depressive-like behaviors.

## Data availability statement

The original contributions presented in the study are included in the article/**Supplementary Material**. Further inquiries can be directed to the corresponding author.

## Ethics statement

The animal studies were approved by Michigan State University Institutional Animal Care & Use Committee and the University of California, San Diego Institutional Animal Care and Use Committee. The studies were conducted in accordance with the local legislation and institutional requirements.

## Author contributions

BV: Conceptualization, Data curation, Formal analysis, Funding acquisition, Investigation, Methodology, Software, Visualization, Writing – original draft, Writing – review & editing. AY: Conceptualization, Data curation, Formal analysis, Funding acquisition, Investigation, Methodology, Visualization, Writing – original draft, Writing – review & editing, Supervision, Validation. JB: Data curation, Investigation, Writing – review & editing. BJ: Data curation, Writing – review & editing. DN: Data curation, Writing – review & editing, Investigation, Methodology. KJ: Data curation, Investigation, Writing – review & editing, Formal analysis. FR: Data curation, Investigation, Writing – review & editing, Conceptualization, Funding acquisition. EH: Investigation, Writing – review & editing. LC: Investigation, Writing – review & editing. DG: Investigation, Writing – review & editing. LS: Investigation, Writing – review & editing, Conceptualization, Data curation, Formal analysis, Funding acquisition, Methodology, Software, Supervision. MG: Investigation, Writing – review & editing, Conceptualization, Funding acquisition, Methodology, Software. KT: Conceptualization, Data curation, Formal analysis, Funding acquisition, Investigation, Methodology, Supervision, Writing – review & editing, Visualization. PM: Conceptualization, Funding acquisition, Methodology, Software, Writing – review & editing, Formal analysis, Supervision, Validation. HH: Conceptualization, Formal analysis, Funding acquisition, Methodology, Software, Supervision, Validation, Writing – review & editing, Data curation, Investigation, Project administration, Resources, Visualization, Writing – original draft.
