# Supplementary material for: The transcription factor VAX1 in VIP neurons of the suprachiasmatic nucleus impacts circadian rhythm generation, depressive-like behavior, and the reproductive axis in a sex-specific manner in mice
Source: Front Endocrinol (Lausanne). 2023 Dec 22;14:1269672. doi: 10.3389/fendo.2023.1269672 (PMC10777845; doi:10.3389/fendo.2023.1269672)
Supplement: Supplementary file 1 [file DataSheet_1.docx]

Supplementary Material

The transcription factor VAX1 in VIP neurons of the suprachiasmatic nucleus impacts circadian rhythm generation, depressive-like behavior, and the reproductive axis in a sex specific manner in mice

Brooke M. Van Loh, Alexandra M. Yaw, Joseph A. Breuer, Brooke Jackson, Duong Nguyen, Krystal Jang, Fabiola Ramos, Emily V. Ho, Laura J. Cui, Dominique L. M. Gillette, Lorenzo Sempere, Michael R. Gorman, Karen J. Tonsfeldt, Pamela L. Mellon, Hanne M. Hoffmann*

***Correspondence:** Corresponding Author: [hanne@msu.edu](mailto:hanne@msu.edu)

# Supplementary Methods

Light composition in the mouse housing and behavioral set-ups were measured with UV Illuminance Spectrophotometer SRI-2000 UV (Allied Scientific Pro^TM^, Québec, Canada). Light measurements were taken with lights ON through the mouse housing cage with the spectrometer sensor facing the light source. The reading for the mouse breeding pairs were analyzed separately, as they were housed on the top shelf of the Optimouse racks, and thus closest to the light source. Behavioral room measurements were taken through the transparent plastic tank used for swim tests. Spectral irradiance was collected from 250 nm to 800 nm in 0.5 nm increments and transferred into the species-specific light exposure calculator (McDowell *et al.*, 2023), as described in (Lucas *et al.*, 2023), allowing to calculate an approximate species specific α-opic equivalent daylight illuminance (EDI). While humans have three different types of cone photoreceptors in their eyes, mice have only two, impacting the mouse’s perception of the color of light. From a human-based spectrometer, values are calculated for each of the mouse’s primary photoreceptors: melanopsin-expressing intrinsically photosensitive retinal ganglion cells (melanopsin), rods, M-cones, and S-cones. These values indicate the perceived tint of the light observed in a species-specific manner. If melanopsin and M-cone α-opic EDI values are equivalent to S-cone EDI values, this indicates light appears similar in color to natural daylight (white). The greater the Melanopsin/M-cone value is as compared to the S-cone value, the more yellow or tinted the light will be perceived by the observer. In contrast, the greater the S-cone value is as compared to the M-cone value, the bluer the light will appear.

## Supplementary Figure


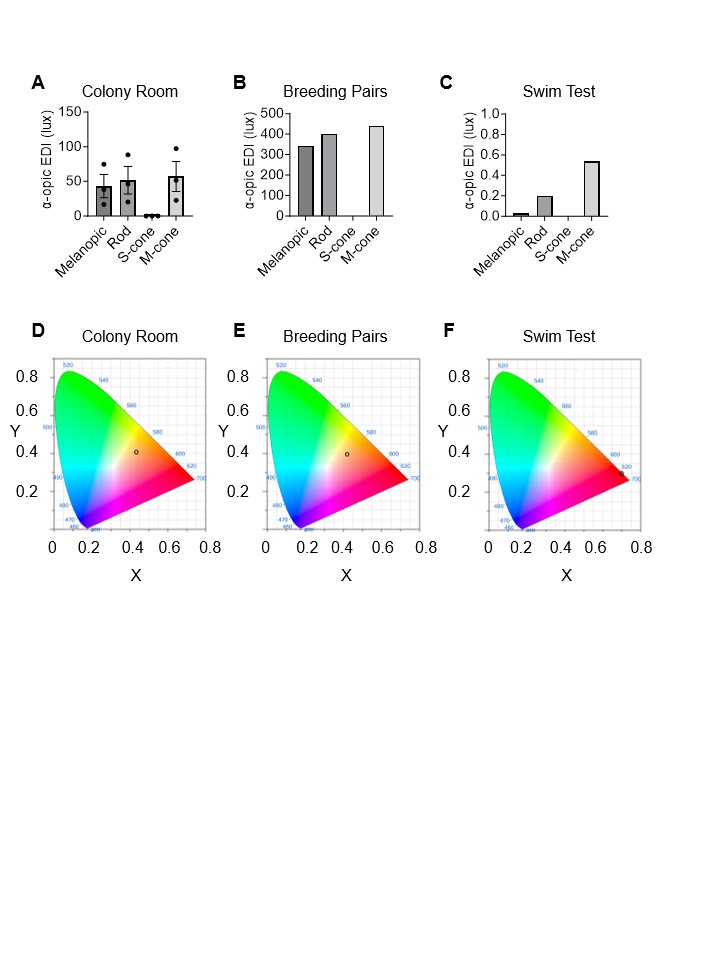


**Supplementary Figure 1. Estimated mouse α-opic equivalent daylight illuminance (EDI). A)** The average α-opic EDI for three different mouse cage locations measured within the colony room, **B)** the cages containing breeding pairs, and **C)** through the swim test equipment in the behavioral room with dim red light. As our measurements show a low s-cone α-opic EDI, the light experienced would appear more yellow to mice compared to the experience of a human. The perceived color of light is important to document, as this may influence mouse behavior and physiology. **D-E)** x,y chromaticity coordinates plotted on commission internationale de l’éclairage (CIE) 1931 color space, as indicated by the small circle, as observed by a human for the indicated mouse locations.

**References**

Lucas, R. J. *et al.* (2023) ‘In the Eye of the Beholder: Measuring and Standardising Light for Laboratory Mammals’, *Preprints*, p. 2023091766. doi: 10.20944/preprints202309.1766.v1.

McDowell, R. J. *et al.* (2023) ‘Beyond Lux: Methods for Species and Photoreceptor-Specific Quantification of Ambient Light for Mammals’, *bioRxiv*. Cold Spring Harbor Laboratory, p. 2023.08.25.554794. doi: 10.1101/2023.08.25.554794.

**
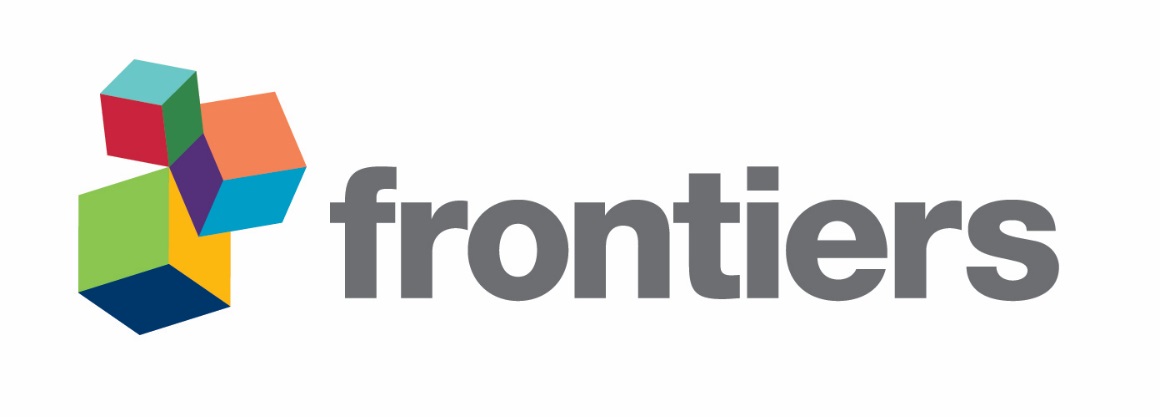
**
